# Editorial: Functional microcircuits in the brain and in artificial intelligent systems

**DOI:** 10.3389/fncom.2023.1135507

**Published:** 2023-01-24

**Authors:** Jung H. Lee, Yoonsuck Choe, Salva Ardid, Reza Abbasi-Asl, Michelle McCarthy, Brian Hu

**Affiliations:** ^1^Pacific Northwest National Laboratory, Seattle, WA, United States; ^2^Department of Computer Science and Engineering, Texas A&M University, College Station, TX, United States; ^3^Department of Applied Physics and Institut d'Investigació per a la Gestió Integrada de Zones Costaneres (IGIC), Universitat Politècnica de València, Gandia, Spain; ^4^Department of Neurology, University of California, San Francisco, San Francisco, CA, United States; ^5^Department of Mathematics and Statistics, Boston University, Boston, MA, United States; ^6^Kitware, Inc., Arlington, VA, United States

**Keywords:** computational modeling, inhibitory neuronal circuit, deep learning, perceptual decision-making, visual perception

Fundamental principles underlying higher-order cognitive functions remain elusive, but recent breakthroughs in neurophysiology and deep learning offer new perspectives. First, experimental studies have uncovered neural circuit motifs consisting of various neuron types; see Brain Initiative Cell Census Network (https://www.nature.com/collections/cicghheddj). For example, inhibitory neuron types expressing exclusive genes have specific targets and distinct functions (Pfeffer et al., 2013). Furthermore, diverse neuron types in cortex and their connectomes were identified in cortical columns (Jiang et al., 2015); see also Barth et al. (2016) for a debate on neuron types. Second, artificial neural networks were originally inspired by structures of the brain (McCulloch and Pitts, 1943) and could be trained to perform complex functions similar to human perception/cognition by deep learning (DL) (Lecun et al., 2015).

## Computational models that can shed light on the links between neural circuits and cognitive functions

“Local” microcircuits, the building blocks of the cerebral cortex of mammalian species, are embedded in larger networks, and thus their functions, rather than being intrinsic, strongly depend on interactions with various other parts within these networks. These intricate network structures pose great challenges when studying the role of local microcircuits in cognition. Computational modeling provides an effective way to study how the local microcircuits contribute to the brain's high-level functions (e.g., perception and decision-making). Lee et al. and Wagatsuma, Shimomura et al. in this Research Topic involve computational modeling focusing on the functional roles of inhibitory neuron types.

One of the essential tasks for visual perception is to distinguish two bordering objects. Border ownership sensitive (BOS) neurons have been known to contribute to this task, but the precise underlying mechanism remains poorly understood. Wagatsuma, Shimomura et al. created a biologically realistic model, which contains somatostatin (SST) and vasoactive intestinal peptide (VIP) expressing inhibitory neurons, to study how selective attention modulates BOS neuron responses. Their simulation results suggest that the disinhibitory control of VIP neurons receiving top-down signals can explain the experimental observation that selective attention enhances the firing rate of BOS neurons but reduces the synchrony between them.

Lee et al. explored potential roles of two major inhibitory neuron types in integrating sensory evidence, which is essential for perceptual decision-making. They built a biologically plausible circuit, in which parvalbumin (PV) and SST expressing inhibitory neurons target specific neuron types in accord with experimental findings. The reported simulation raised the possibility that the brain uses the location of highly active neurons (“bump activity”) in the cortical networks to store sensory evidence. Specifically, the model has two modes, the integration mode, in which the bump activity propagates, and the retention mode, in which the bump activity remains stationary. With these two modes, the newly proposed model can integrate and retain sensory evidence, and SST inhibitory neurons are responsible for switching between these two modes.

## Synergistic relationships between deep learning and brain science

DL has been developed to perform complex functions similar to our cognition. DL's notable success in multiple domains including computer vision and language models suggests that deep neural networks (DNNs) and the brain could rely on similar mechanisms when processing sensory inputs. With this possibility in mind, Wagatsuma, Hidaka et al. compared the internal representations of monkey visual cortices and AlexNet [a Convolutional Neural Network (CNN), a type of DNN]. Neural responses in the earlier layers of AlexNet were correlated with those of the primary visual cortex, whereas the responses in later layers were correlated either with those of V4 or the inferior temporal cortex, supporting the possibility that the brain and DNNs rely on similar hierarchical signal processing for visual object classification and detection. This work is representative of how advances in neuroscience can help building more efficient DL algorithms, as well as how advances in DL can help to better understand brain circuit function and operation.

Emerging “NeuroAI” seeks to leverage biological mechanisms to improve DL (Zador et al., 2022), and a line of studies (see Clopath et al., 2010, for example) uses biological spike-time-dependent plasticity (STDP) to train spiking neural networks (SNNs), a form of power-efficient biological neural networks. Haşegan et al. proposed a novel evolutionary strategy for SNNs, which is more efficient than traditional STDP-based learning algorithms when training a SNN to play a cart-pole game. As the building blocks of the brain, SNNs can naturally be better platforms for NeuroAI than current DNNs are. However, SNNs utilize discrete “spikes” for their functions, and backpropagation (the backbone of DL) cannot be directly used to train them, suggesting the need for stronger collaboration between neuroscience and deep learning communities.

## Author contributions

All authors listed have made a substantial, direct, and intellectual contribution to the work and approved it for publication.

## References

[B1] Barth A. Burkhalter A. Callaway E. M. Connors B. W. Cauli B. DeFelipe J.. (2016). Comment on “Principles of connectivity among morphologically defined cell types in adult neocortex”. Science 353, 1108. 10.1126/science.aaf5663 27609882

[B2] Clopath C. Büsing L. Vasilaki E. Gerstner W. (2010). Connectivity reflects coding: a model of voltage-based STDP with homeostasis. Nat. Neurosci. 13, 344–352. 10.1038/nn.2479 20098420

[B3] Jiang X. Shen S. Cadwell C. R. Berens P. Sinz F. Ecker A. S.. (2015). Principles of connectivity among morphologically defined cell types in adult neocortex. Science 350, aac9462. 10.1126/science.aac9462 26612957 PMC4809866

[B4] Lecun Y. Bengio Y. Hinton G. (2015). Deep learning. Nature 521, 436–444. 10.1038/nature14539 26017442

[B5] McCulloch W. S. Pitts W. (1943). A logical calculus of the ideas immanent in nervous activity. Bull. Math. Biophys. 5, 115–133. 2185863

[B6] Pfeffer C. K. Xue M. He M. Huang Z. J. Scanziani M. (2013). Inhibition of inhibition in visual cortex: the logic of connections between molecularly distinct interneurons. Nat. Neurosci. 16, 1068–1076. 10.1038/nn.3446 23817549 PMC3729586

[B7] Zador A. Richards B. Ölveczky B. Escola S. Bengio Y. Boahen K.. (2022). Toward next-generation artificial intelligence: catalyzing the NeuroAI revolution. arXiv preprint arXiv:2210.08340. 10.48550/arXiv.2210.08340

